# Unique induction of p21^WAF1/CIP1^expression by vinorelbine in androgen-independent prostate cancer cells

**DOI:** 10.1038/sj.bjc.6601317

**Published:** 2003-10-14

**Authors:** X M Liu, J D Jiang, A C Ferrari, D R Budman, L G Wang

**Affiliations:** 1 Department of Medicine, Division of Medical Oncology, Mount Sinai School of Medicine, One Gustave L Levy Place, Box 1129, New York, NY 10029, USA; 2 North Shore University Hospital, New York University School of Medicine, 300 Community Drive, Manhasset, NY 11030, USA

**Keywords:** androgen-independent cell, p21 ^WAF1/CIPI^-deficit cell, p21 ^WAF1/CIP1^induction, vinorelbine and paclitaxel

## Abstract

To study the mechanisms of the development of hormone refractory prostate cancer, we established an androgen-independent (AI) prostate cancer cell line derived from hormone-dependent (AD) LNCaP cells. Our previous studies have demonstrated that AI cells are deficient in expression of p21^WAFl/CIP1^ (p21) due to overexpressed AR and are resistant to apoptosis. In this study, the induction of p53 and p21 expression by vinorelbine (Navelbine) was compared between AD and AI cells in an attempt to understand the difference(s) in apoptotic signalling pathways in these cells. Using a series of deletion of p21 reporter constructs, we found that vinorelbine mediated p21 induction in a p53-dependent manner in AD cells. In contrast, p21 expression restored by vinorelbine in AI cells was found to be through both p53-dependent and-independent pathways. In the absence of two p53 binding sites, Spl-3 and Spl-4 sites, in the promoter of human p21 gene, were found to be required for vinorelbine-mediated p21 activation. No p21 induction was observed by paclitaxel in AI cells. Exposure of AI cells to paciltaxel followed by vinorelbine produced synergism. Our data, thus, provide a basis for the synergistic combination of vinorelbine and paclitaxel for the treatment of advanced prostate cancer.

Vinorelbine is a third-generation vinca alkaloid that plays an important role in the treatment of several human cancers, including breast carcinoma, cancers of ovarian, prostate, and lung as well as haematological malignancies (Budman, 1997; Trimble, 2000; Lilenbaum, 2001). In contrast to paclitaxel, vinorelbine binds microtubules and prevents their polymerisation, thus interfering with mitosis (Krikorian and Breillout, 1991). Previous studies have demonstrated that independent of the type of effect by the antimicrotubule agent on microtubule assembly (polymerisation or depolymerisation), these drugs exhibit the ability to promote apoptosis in cancer cells (Wang *et al*, 1999b,^2000^,^2001^; Budman *et al*, 2000; Charles *et al*, 2001; Fan *et al*, 2001; Liu *et al*, 2001). One of mechanisms of vinorelbine-induced apoptosis is thought to be the upregulation of p21 and p53. Expression of p21 is regulated through p53-dependent and -independent pathways (Blagosklonny *et al*, 1995; Barboule *et al*, 1997; Torres and Horwitz, 1998; Wang *et al*, 1999b).

Since the observations by Huggins and Hodges (1941) in the early 1940s on the benefit of androgen ablation, no major therapeutic advances have been achieved for prostate cancer patients exhibiting hormone independence, disease metastatic to regional lymph nodes or those with more distant metastases. The majority of patients succumb to the growth of androgen-independent disease within 6–18 months. To understand the mechanisms of development of hormone refractory prostate cancer, we established an androgen-independent (AI) prostate cancer cell line derived from the hormone-dependent (AD) LNCaP cells that lack p2l^WAF1/CIP1^ (p21) expression and are resistant to the induction of apoptosis (Gao *et al*, 1999). Our recent studies demonstrate that silencing of p21 in AI cells is linked to the overexpression of the AR in this cell line (Wang *et al*, 2001). In the present study, we explore whether the microtubule targeting agents, vinorelbine and paclitaxel, could induce p21 expression in AI cells. We demonstrate that vinorelbine, but not paciltaxel, is able to restore p21 expression of AI cells through both p53-dependent and -independent pathways.

## MATERIALS AND METHODS

### Reagents

Monoclonal antibodies against p21, p53 and *β*-actin, as well as secondary antibodies were purchased from DAKO Corporation (Carpinteria, CA, USA) and Santa Cruz Biotechnology, Inc. (Santa Cruz, CA, USA), respectively. Western blotting detection reagents and ECL films were purchased from Amersham Pharmacia Biotech Inc. (Buckinghamshire, England). Effectene transfection reagent, and luciferase assay kit, pSV-*β*-galactosidase control plasmid and its assay kit were obtained from Qiagen (Valencia, CA, USA) and Promega (Madison, WI, USA), respectively. Vinorelbine was provided by Glaxo SmithKline Inc. (Research Triangle Park, NC, USA). Trichostatin A (TSA), taxol (paclitaxel) and other chemicals were purchased from Sigma-Aldrich (St Louis, MO, USA).

### p21 constructs

The full p21-promoter luciferase reporter construct was a gift from Dr Lewis Silverman (Mount Sinai School of Medicine, New York, USA), and p21 reporters pWPdel-BstX I, pWPlOl, pWPdel-Sma I, Spl-luc and mtSpl-luc were kindly provided by Dr Yoshihiro Sowa (University of Medicine, Kamigyo-ku, Kyoto 602, Japan). The full-length promoter is a 2.4-kb genomic fragment subcloned into the *Hin*dIII site of the luciferase reporter vector, pGL2-basic (Promega, Madison, WI, USA) (Datto *et al*, 1995). The pWPdel-BstX I(−133 to +1 relative to the start site) contains all six Sp-1 sites without the p53 binding site (Nakano *et al*, 1997; Sowa *et al*, 1997). The pWPlOl, by deleting Spl-1 and Spl-2 from pWPdel -BstXI, consists of a minimal promoter region of 101 relative to the transcriptional start site containing four Spl binding sites, termed Spl-3, Spl-4 Spl-5, and Spl-6. pWP-del-SmaI was generated by further deletion of Spl-3 and Spl-4 from pWPlOl, which contains only two Spl sites, Spl-5 and Spl-6. The Spl-luc and mtSpl-luc were reporters of luciferase promoted by three tandems of repeats of consensus Spl sites and mutant Spl sites.

### Cell culture

LNCaP cells were purchased from the American Type Culture Collection (Rockville, MD, USA) and maintained in RPMI-1640 (GIBCO, Gaithersburg, MD, USA) containing 10% heat-inactivated bovine serum (FBS). The AI LNCaP subline derived from LNCaP cells was maintained in RPMI-1640 medium containing 10% charcoal-stripped, heat-inactivated FBS (CSFBS) (Hyclone Laboratories, Inc., Logan, UT, USA) and 5 *μ*g ml^−1^ of insulin as described previously (Gao *et al*, 1999).

### Western blotting of cellular protein extraction

Total cellular proteins were extracted from AD or AI cells under the various treatment conditions as indicated, and Western blotting assay was performed as described previously (Wang *et al*, 1999a). Briefly, 50 or 100 *μ*g cellular extracts were separated by 4/10% stack SDS–PAGE, electro-transferred to nitrocellulose filters, and immunoblotted with monoclonal antibodies against p53 and p21 or *β*-actin, respectively. Quantitation by densitometry of the ECL films was performed using an Imaging Densitometer Model GS-720 (Bio-Rad Lab. Hercules, CA, USA) and normalised to the level of *β*-actin.

### DNA transfection and luciferase activity assay

DNA transfection and luciferase activity assays were performed as described previously with modifications (Wang *et al*, 1999a). Androgen-dependent and AI cells in exponential growth in regular serum medium in 60-mm dishes were washed once with serum-free mammary epithelium growth medium (MEGM) and reincubated in the MEGM. The cells were then transfected with 1 *μ*g cDNA of p21-luciferase reporter and 0.5 *μ*g of *β*-galactosidase control vector DNA using Effectene according to the manufacturer's instructions. At 1 day after the transfection, the cells were exposed for an additional 24 h to various concentrations of vinorelbine or paclitaxel as indicated. The cells were washed, lysed, and the lysis simultaneously assayed for activities of luciferase and of *β*-galactosidase using the Promega assay systems according to the manufacturer's instructions. The luciferase activity, normalised to galactosidase, was expressed as units per milligram of protein.

### Combination analysis

Exponentially growing LNCaP cells were planted in 96-well dishes at a density of 5000 cells per well. At 24 h after the incubation, the cells were exposed to series dilution of paclitaxel, vinorelbine for 7 days or sequentially exposed to paclitaxel for 3 days, followed by paclitaxel plus vinorelbine for an additional 4 days at maximal concentration of 50 nM, respectively. After the incubation, the cell growth was measured by MTT as described previously (Kreis *et al*, 1997), and the percentages of inhibition (1-*T*/*C*%) were calculated, and the data were analysed by a PC program, *CalcuSyn*, of Biosoft edited by T-C Chou, Memorial Sloan-Kettering Cancer Center, New York, and MP Hayball, at Cambridge, UK, 1996 (Wang *et al*, 1997), and *Sigma Plot.* Regression and statistical analysis were performed using *Sigma Plot* program. Combination indexes (CI) calculated by *CalcuSyn* were used to evaluate the outcomes of the combination. Outcomes of the combinations were determined by CI. If CI >1, the combination is antagonistic, CI=1, additive and CI <1, synergistic.

## RESULTS

### Kinetics of AD and Al cell growth

**Figure 1:**
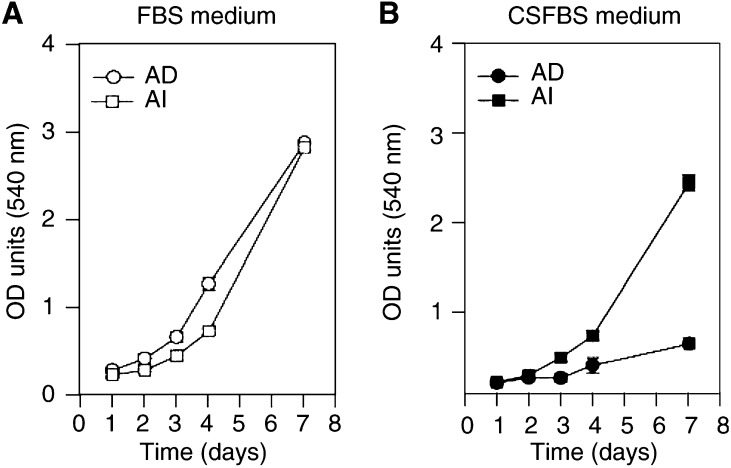
Typical kinetic curves of AD and AI cell growth in the presence or absence of androgens. AD and AI cells grown exponentially were harvested and reincubated in 96-well dish at a density of 2500 cells per well in 200 *μ*l of RPMI-1640 medium containing 10% FBS (panel **A**) or 10% CSFBS (panel **B**). The cells were maintained in 5% CO_2_ incubator at 37°C for different periods of time as indicated. The growth of cells was then measured by MTT as described in the ‘Materials and Methods’.

To study the mechanisms of the progression of hormone refractory prostate cancer, we established an AI prostate cancer cell line derived from AD LNCaP cells and demonstrated that Al cells are resistant to apoptosis and are deficient in expression of p21, which at least in part is due to the overexpressed AR (Gao *et al*, 1999; Wang *et al*, 2001). Typical kinetic growth curves, as measured by MTT, of those two cell lines in the presence or absence of androgen are shown in Figure 1

. No significant difference in growth rate between AD and AI cells was observed when both cell lines were incubated in the RPMI-1640 containing androgens (10% FBS; Figure 1, left panel). However, growth of AD cells became much slower as compared with their successors when the cells were grown in the same medium containing no androgens (10% CSFBS; Figure 1, right panel), confirming that androgens are required for the growth of the androgen-dependent cells. A similar growth rate was obtained when AI cell were fed with full serum medium as compared with the coal-stripped serum, demonstrating that the cells become AI (Figure 1, left and right panels).

### Induction of p53 and p21^WAFI/C1P1^ expression by vinorelbine in AD and AI cells

**Figure 2:**
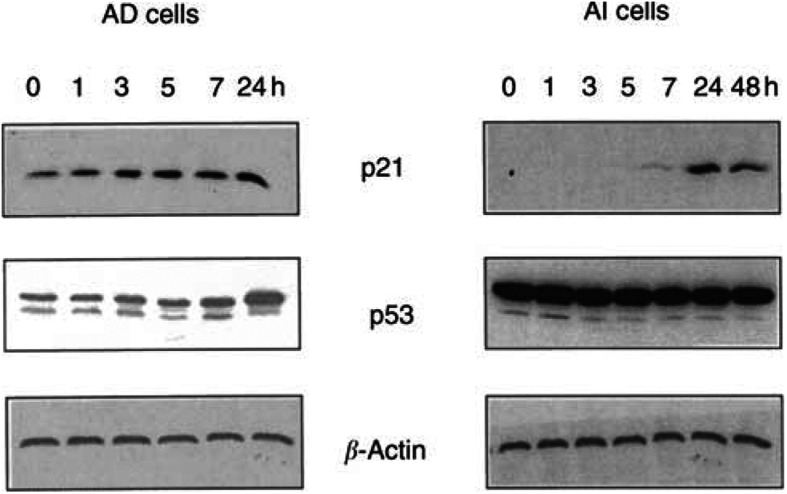
Time-dependent induction of p53 and p21^WAFI/CIP1^ expression in androgen-dependent (AD) and- independent (AI) prostate cancer cell lines by vinorelbine. Androgen-independent and AI cells grown exponentially were exposed to 0.1 *μ*M of vinorelbine for indicated periods of time. The cells were harvested, washed, and total proteins extracted. Proteins (50 *μ*g) were subjected to 4/10% stacking SDS–PAGE, electrotransferred to nitrocellulose membrane, and immunoblotted with p21^WAFI/CIP1^ and p53 monoclonal antibodies, respectively. The same membrane was stripped, and reprobed with antibody against *β*-actin for equal loading control.

Induction of p53 and p21 by antimicrotubule agents has been considered to be one mechanism involved in the apoptotic process in cancer cells (Blagosklonny *et al*, 1995; Barboule *et al*, 1997; Torres and Horwitz, 1998; Wang *et al*, 1999b). To explore whether vinorelbine and paclitaxel are able to induce p21 expression in AD and AI cells that are deficient in p21, and the possible differences between AD and AI cells in expression of p53 and p21 in response to treatments with vinorelbine or paclitaxel, the resultant levels of p53 and p21 were determined by Western blotting. As shown in Figures 2

and 3

**Figure 3:**
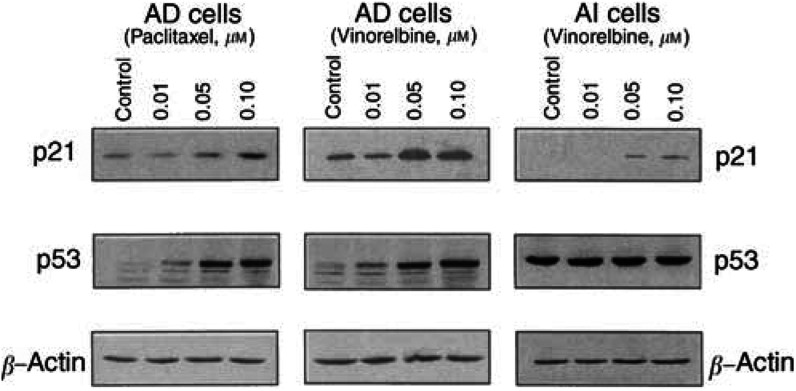
Effects of paclitaxel (left panel) and vinorelbine (middle and right panel) on the expression of p53 and p21^WAFI/CIPI^ in androgen-dependent (AD, left and middle panel) and-independent (AI, right panel) prostate cancer cell lines. AD and AI cells grown exponentially were exposed for 24 h to different concentrations of paclitaxel (left panel) or vinorelbine (middle and right). The cells were harvested, washed, and total proteins extracted. Fifty *μ*g of proteins were subjected to 4%/10% stacking SDS-PAGE, electrotransferred to nitrocellulose membrane, and immunoblotted with p21^WAFI/CIPI^ and p53 monoclonal antibodies, respectively. The same membrane was stripped and reprobed using antibody against *β*-actin for equal loading control.

, exposure of AD cells to vinorelbine resulted in concentration- and time-dependent increases in the protein levels of both p53 and p21. The amount of p53 protein increased approximately 23% 3 h after the exposure of AD cells to 0.1 *μ*M of vinorelbine (Figure 2, left panel). The maximal induction (over two-fold) of the protein by 0.05 *μ*M of vinorelbine was achieved at 24 h (Figure 3, middle panel). The increase of p21 protein paralleled the elevated protein level of p53, indicating that the p21 induction by vinorelbine is a p53-dependent process in AD cells.

Consistent with our previous observation (Gao *et al*, 1999) no p21 protein was detected in untreated AI cells, as shown in Figure 2, right panel. However, the p21 protein became detectable 7 h after exposure of the AI cells to vinorelbine. In contrast to AD cells, constant high levels of p53 proteins were observed in AI cells that did not respond to vinorelbine.

Treatment of AD cells with various concentrations of paclitaxel also significantly induced p53 expression and resulted in a concentration-dependent increase in the level of p21 protein (Figure 3, left panel). However, no significant changes in expression of those proteins were observed in AI cells under the same experimental conditions (data not shown).

**Figure 4:**
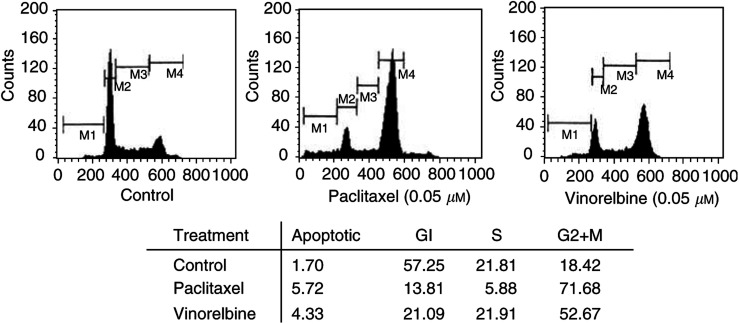
Cell cycle distribution of AI cells after exposure to paclitaxel or vinorelbine. Androgen-independent cells at exponential growth phase were exposed to 0.05 *μ*M of paciltaxel (middle panel) or vinorelbine (right panel) for 24 h. The cells were harvested, washed with cold PBS, fixed with 70% ethanol and propidium iodide (PI) stained. The cell cycle distribution was then analysed by FACScan (Beckton Dickinson, Mountainview, CA, USA).

Analysis of cell cycle distribution showed higher percentage of cells (21.09%) at Gl phase when AI cells were exposed to vinorelbine compared to cells exposed to paclitaxel (13.81%, Figure 4

), probably due to the expression of p21 induced by vinorelbine in this cell line.

**Figure 5:**
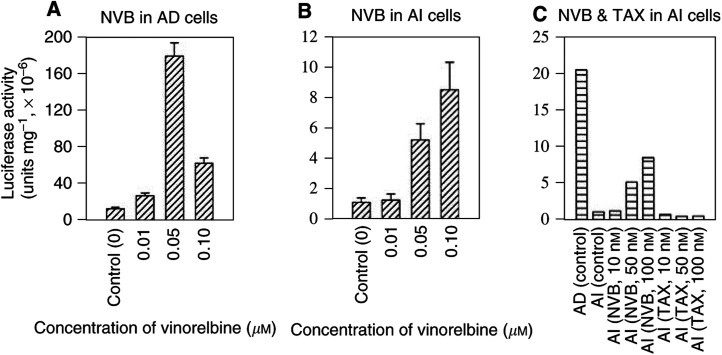
Effect of vinorelbine (NVB) or paclitaxel (TAX) on p21 transactivation in AD or AI cells. Androgen-dependent or AI cells grown at exponential phase were cotransfected in serum-free RPMI-1640 medium with 1 *μ*g cDNA of full promoter-p21 reporter and 1 *μ*g cDNA of pSV-*β*-galactosidase using Effectene according to manufacturer's instructions. At 1 day after the transfection, the cells were exposed for 24 h to concentrations of NVB or TAX, as indicated. The cells were washed, and lysed for luciferase activity assay as described in Materials and Methods. The luciferase activities in AD or AI cells were normalised to the activity of *β*-galactosidase for the equal efficiency of the transfections. The data are expressed as means±s.d. from three separate experiments.

To examine whether vinorelbine stimulates p21 expression through its promoter transcriptional activation, a p21 luciferase reporter construct containing the full-length of human p21 promoter was transiently transfected into both AD and AI cells. At 1 day after the transfection, the cells were either exposed for 24 h to different concentrations of vinorelbine, or vehicle as controls. As shown in Figure 5

, the luciferase activity was significantly induced by vinorelbine (*P*<0.01) in both AD (panel A) and AI (panel B) cells. Maximal induction in AD cells was achieved at concentration of 0.05 *μ*M with approximately 14-fold increase of the luciferase activity (*P*<0.01). Higher concentration (0.1 *μ*M) of the treatment reduced the extent of the induction, possibly due to cytotoxicity (Figure 5, panel A). Induction of reporter activity in AI cells occurred at concentration of 0.05 *μ*M, and achieved maximal induction at concentration of 0.10 *μ*M, almost a nine-fold increase (*P*<0.01). In contrast to vinorelbine, paciltaxel did not stimulate the p21 reporter activity in AI cells under the same experimental conditions (panel C). These findings paralleled the results obtained by Western blotting assay.

### Vinorelbine-mediated p21 induction in AI cells through p53 and Spl pathways

**Figure 6:**
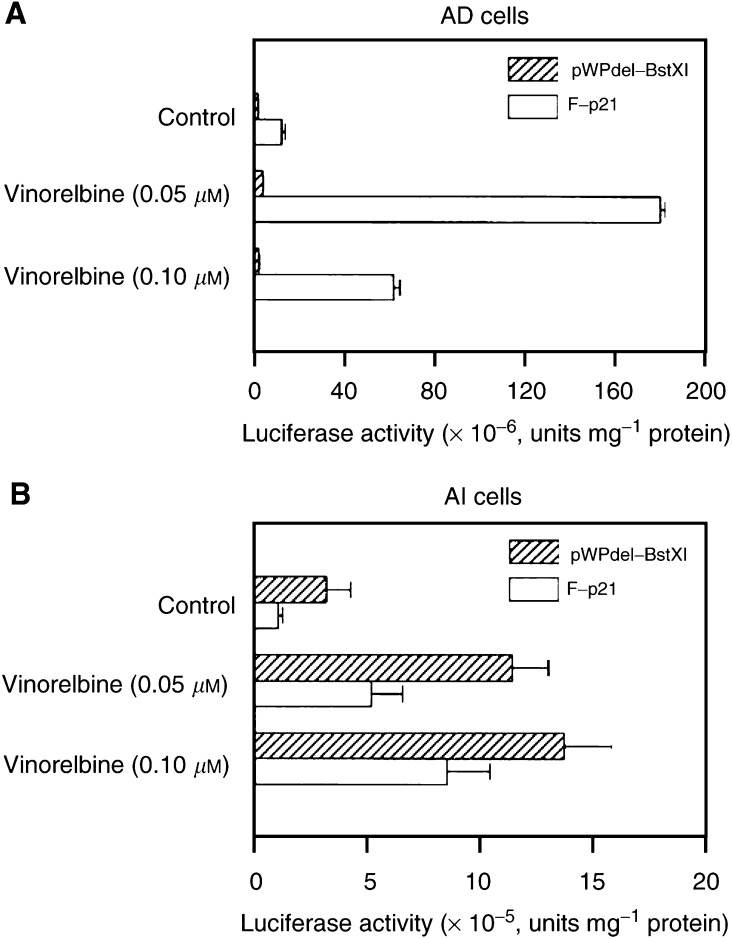
Role of p53 binding sites within the promoter of p21 gene played in the vinorelbine-mediated p21 activation in AD and AI cells. Androgen-dependent and AI cells grown at exponential phase were cotransfected in serum-free RPMI-1640 medium with 1 *μ*g cDNA of full promoter-p21 reporter or pWPdel-BstXI and 1 *μ*g cDNA of pSV-*β*-galactosidase using Effectene according to the manufacturer's instructions. At 1 day after the transfection, the cells were exposed for 24 h to concentrations of vinorelbine as indicated. The cells were washed and lysed for luciferase activity assay as described in Materials and Methods. The luciferase activities in AD and AI cells were normalised to the activity of *β*-galactosidase for the equal efficiency of the transfections. The data are expressed as means± s.d. from three separate experiments.

To define the role p53 played in the vinorelbine-mediated upregulation of p21 expression, we compared the stimulation effects of vinorelbine on the full p21 promoter reporter and the reporter without p53 regulatory binding sites (pWPdel-BstXI). As shown in Figure 6

, the basal level of the transcriptional activity of the full promoter of p21 reporter gene in AD cells was found to be four-fold higher than that of pWPdel-BstXI (*P*<0.01). In AI cells, the basal activity of pWPdel-BstXI was 1.5-fold higher than that of F-p21. Although a substantial increase in F-p21 activity (approximately 14-fold) was observed when the AD cells were exposed to 0.05 *μ*M of vinorelbine, no such induction for pWPdel-BstXI activity was obtained in AD cells under the same experimental condition (Figure 6, panel A).

In contrast to AD cells, as shown in Figure 6, panel B, both reporter genes in AI cells were similarly and significantly stimulated by vinorelbine in a concentration-dependent manner, although the extent of the induction of those reporter activities by vinorelbine is different in AI cells. The maximal induction of F-p21 reporter activity (nine-fold) was achieved at concentration of 0.10 *μ*M of vinorelbine (*P*<0.01), whereas only a 4.5-fold increase of pWPdel-BstXI activity was obtained under the same experimental conditions.

**Figure 7:**
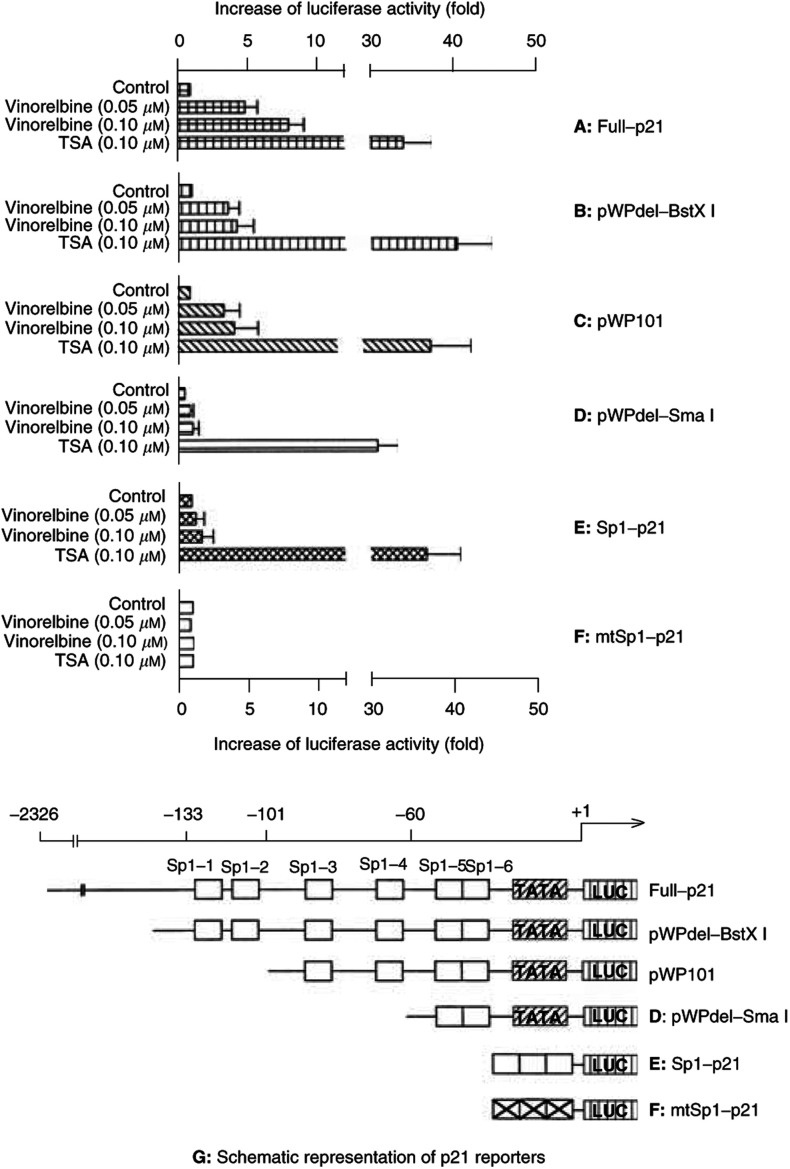
Involvement of Spl-3 and Spl-4 within the promoter of p21 gene in the vinorelbine-mediated p21 transcriptional activation in androgen-independent prostate cancer cells. Androgen-independent cells grown at exponential phase were cotransfected in serum-free RPMI-1640 medium with 1 *μ*g cDNA of full promoter p21 reporter, pWPdel-BstXI, pWPlOl, pWPdel-Sma I, Spl-p21, or mtSpl-p21 and 1 *μ*g cDNA of pSV-*β*-galactosidase using Effectene according to the manufacturer's instructions. At 1 day after the transfection, the cells were exposed for 24 h to different concentrations of vinorelbine, as indicated. The cells were washed, and lysed for luciferase activity assay as described in Materials and Methods. The luciferase activities in AD and AI cells were normalised to the activity of *β*-galactosidase for the equal efficiency of the transfections. The data are expressed as means±s.d. from three separate experiments.

Recent data have indicated that p21 expression can be induced by histone deacetylase inhibitors through p53-independent Spl sites of the promoter of the gene (Sowa *et al*, 1997). To evaluate whether vinorelbine-mediated induction of p21 in AI cells followed a similar mechanism analogous to histone deacetylase inhibitors, transfection assays were carried out using p21 reporter plasmids containing different lengths of Spl sites, or their mutated forms. As shown in Figure 7

, p21 reporter with the complete promoter showed highest inducible activity (over nine-fold increase) by vinorelbine. In contrast, approximately a 4.5-fold increase of luciferase activity induced by vinorelbine was obtained when the reporter contained only complete Spl sites (pWPdel-BstXI). Similar transcriptional activation was obtained using pWPlOl in which Spl-1 and Spl-2 were deleted, indicating that those two Spl sites were not essential for vinorelbine-mediated p21 activation. Of note, under the same experimental conditions only approximately a two-fold increase (*P*<0.05) of the activity was observed in pWPdel-SmaI reporter containing only Spl-5 and Spl-6. Moreover, if the reporter contained just three tandem repeats of consensus Spl sites (Spl-p21), no significant induction of p21 activity by 0.1 *μ*M of vinorelbine (1.74-fold, *P*>0.05) was observed, while a significant increase was achieved by 0.1 *μ*M of TSA, a well-established histone deacetylase inhibitor.

### Synergistic combination of paclitaxel and vinorelbine

**Figure 8:**
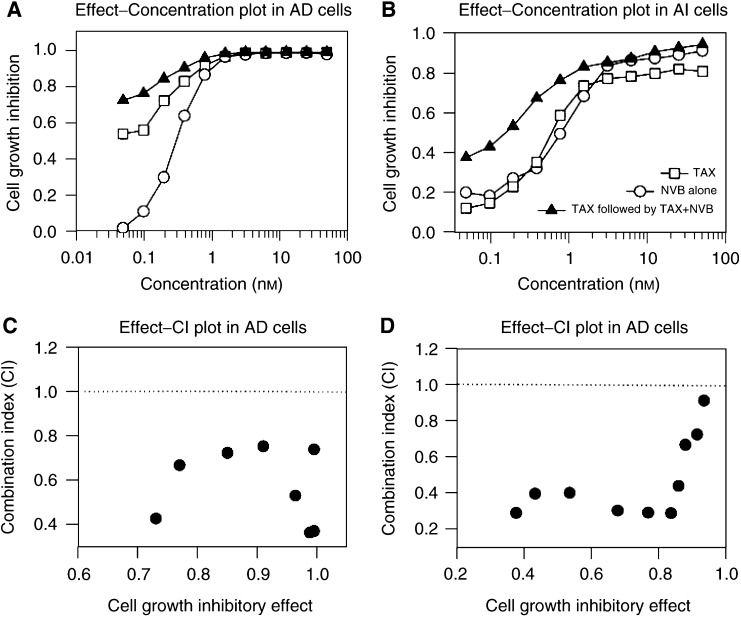
Synergistic effects of sequential combination of paclitaxel and vinorelbine in AD and AI cells. Androgen-dependent and AI cells at exponential growth phase in 96-well dishes were exposed to a series dilution of either paclitaxel (TAX) or vinorelbine (NBV) alone for 7 days or paclitaxel for 3 days followed by paclitaxel plus vinorelbine for additional 4 days. Cell growth was determined by MTT, and the combination indexes (CI) were calculated by a PC program *CalcuSyn* as described in the Materials and Methods. Panels **A** and **B**, the effect–concentration plots in AD (**A**) and AI (**B**); Panels **C** and **D**, the effect –combination index plots in AD (**C**) and AI (**D**). Panel **E**: the expression of p53 and p21 in AD and AI cells after exposure for 24 h to NVB or TAX alone or NVB plus TAX.

It has been reported that flavopridol, an inhibitor of cyclin-dependent kinases, significantly enhances paclitaxel-induced apoptosis when paclitaxel precedes flavopridol (Schwartz *et al*, 2002). Since vinorelbine, but not paclitaxel, was able to induce p21 expression in AI cells, we postulated that the sequential exposure of AD and AI cells to paclitaxel followed by vinorelbine might produce synergistic outcomes. To explore this possibility, we treated the cells with either paclitaxel or vinorelbine alone for 7 days, or paclitaxel for 3 days followed by paclitaxel plus vinorelbine for additional 4 days, and analysed for the outcomes of the sequential combination by a PC program *CalcuSyn* (Kreis *et al*, 1997). As expected, this combination resulted in a significant synergy in both AD and AI cells (Figure 8

). All combination indexes were smaller than 1 (Figure 8, panels B and D). However, on concurrent exposure of AI cells to paclitaxel plus vinorelbine, the calculated combination indexes were found to be between 1.5 and 3.8, suggesting an antagonistic to moderate additive effects (data not shown). The calculated IC_50_ under the described experimental conditions were shown in Table 1

**Table 1:** IC_50_ in AD and AI cells in response to the treatment of either paclitaxel or vinorelbine alone for 7 days, or paclitaxel for 3 days followed by vinorelbine for additional 4 days

**Cell lines**	**Treatment**	**IC_50_ (nM)**	**P-value**
AD	Paclitaxel (T)	0.035±0.005	T*vs* N, *P*<0.01
AD	NVB (N)	0.310±0.061	T *vs* T+N, *P*<0.001
AD	Paclitaxel followed by T + N	0.0015±0.0007	N *vs* T+N, *P*<0.001
AI	Paclitaxel (T)	0.650±0.140	T *vs* N, *P*>0.05
AI	NVB (N)	0.590±0.091	T *vs* T+N, *P*<0.001
AI	Paclitaxel followed by T + N	0.110±0.003	N *vs* T+N, *P*<0.001

The IC_50_ was calculated and statistically analysed between the treatments using PC program *Sigma Plot* from three separate experiments. T=treated with paclitaxel, N=treated with vinorelbine (NVB), and T+N=treated with paclitaxel followed by paclitaxel plus NVB as described in ‘Materials and Methods’.

in which the IC_50_ from the combination were significantly smaller than with either paclitaxel or vinorelbine alone (*P*<0.001) in both AD and AI cells. In addition, while the sensitivity of AD cells to paclitaxel (IC_50_ 0.035 nM) was almost 10-fold higher than that of vinorelbine (0.31 nM, *P*<0.001), no significant difference was observed in AI cells (0.65 nM of paclitaxel *vs* 0.59 nM of vinorelbine, *P*>0.05).

## DISCUSSION

Using a series deletion of p21 reporter constructs, we have demonstrated that vinorelbine induced p21 expression in AI cells in both p53-dependent and -independent manners. While nine-fold induction of activity of full promoter-p21 reporter was achieved at concentration of 0.10 *μ*M of vinorelbine (*P*<0.01), only a 4.5-fold increase in activity of pWPdel-Bst XI reporter (only contains six Spl binding sites) was obtained under the same experimental conditions. Deletion of Spl-1 and Spl-2 from pWPdel-Bst XI to generate pWPlOl did not result in further reduction of the transcriptional activation (4.5-fold), suggesting that Spl-1 and Spl-2 within the p21 promoter was not essential for vinorelbine-mediated p21 activation. This effect is distinct from the effects of phorbol ester and of okadaic acid (Biggs *et al*, 1996). However, using the reporter pWPdel-Sma that contains Spl-5 and Spl-6, the expression only achieved approximately a two-fold induction (*P*<0.01). Moreover, activity of three tandem repeats of consensus Spl-driven reporter (Spl-luc) was only induced 1.7-fold by vinorelbine, whereas it was induced over 30-fold by TSA, which was consistent with previous report (Sowa *et al*, 1997). These findings indicate that in addition to two p53 regulatory sites, Spl-3 and Spl-4 in the promoter of human p21 gene are required for vinorelbine-mediated transcriptional activation of p21 in the p21-deficient AI cells, and this induction is distinct from that seen with histone deacetylase inhibitors.

Our study also demonstrated that vinorelbine failed to induce transcriptional activation of p21 reporters without p53 binding sites in AD cells. One possible interpretation of this result may be that in the presence of active p53, vinorelbine-mediated p21 induction is preferentially achieved through a p53-dependent pathway as indicated by significant increase in the levels of p53 proteins (Figure 1), and the Spl pathway is activated when p53 activity is not present. The p53 proteins in AI cells were constantly maintained at a high level and no changes were observed in response to the treatment with vinorelbine (Figures 2 and 3), suggesting an abnormality present in the product of this tumour suppressor gene.

Paclitaxel has been reported to induce concentration- and time-dependent accumulation of p21 in both p53 wild-type MCF-7 and p53-null PC3 M cells (Blagosklonny *et al*, 1995; Barboule *et al*, 1997). In this study, we demonstrated that paclitaxel failed to induce p21 expression in AI cells. A similar observation has also been reported in that paclitaxel could not induce p21 expression in NIH-OVCAR-3 cells that are deficient in basal expression of p21. Therefore, these data indicate that paclitaxel may be unable to induce p21 expression in cells that are deficient in basal expression of the gene. The obvious consequence is that such cells may be resistant to apoptotic induction by paclitaxel, which was supported by this study in that the sensitivities of AD and AI in response to paclitaxel were significantly different from that to vinorelbine. This conclusion is also supported by the fact that increased p21 level usually parallels induction of apoptosis mediated by chemotherapeutic agents, including taxanes (Ali *et al*, 2003; Ganansia-Leymarie *et al*, 2003; Lee *et al*, 2003). Paclitaxel was able to induce p21 expression in AD cell, thus producing greater cytoxicity (IC_50_ 0.035 nM in AD as compared 0.61 nM in AI cells). In contrast, vinorelbine was able to induce p21 expression in both AD and AI cells, hence, only a small difference in the sensitivity was observed between those cell lines (IC_50_ in AD 0.31 *vs* 0.59 nM in AI cells).

Alterations in expression of cell cycle regulators, such as E2F-1, cyclin/Cdks (cyclin Dl/Cdk4, cyclin A/Cdk2), and cyclin-dependent kinase (Cdk) inhibitors (p6, p21, and p27), play an important role in regulation of drug sensitivity (Hochhauser *et al*, 1996; St Croix *et al*, 1996; Li *et al*, 1997). NIH-OVCAR-3 cells that are deficient both in basal- and paclitaxel-induced p21 are associated with apoptotic resistance (Barboule *et al*, 1997). We have previously demonstrated that loss of p21 expression in our newly established AI cells may play an important role in apoptotic resistance (Gao *et al*, 1999). Therefore, restoration of normal expression of those cell cycle modulators may allow cells to regain apoptotic sensitivity. We recently demonstrated that AI cells exposed to TSA overcame their resistance to apoptosis induced by paclitaxel, probably due to transcriptional activation of p21 by TSA (Sowa *et al*, 1997; Wang *et al*, 2001). In this report, we demonstrated that vinorelbine, but not paclitaxel, was able to restore p21 expression of AI cells. Our findings, thus, may provide a theoretical basis for the synergistic combination of vinorelbine and paclitaxel for the treatment of advanced prostate cancer. The significant synergistic effects produced by sequential exposures of both AD and AI cells to paclitaxel followed by paclitaxel plus vinorelbine supported this hypothesis.

Expression of p21 has been demonstrated to be regulated through both p53-dependent and-independent pathways (Cartel and Tyner, 1999). Transcriptional activation of p21 triggered by DNA damage was found to be present in a p53-dependent manner in most tissues/cells acting by two p53- binding sites located in promoters -2301 and -394 of p21 gene. p21 expression induced by other factors, that is, Zta, NDF, c-Rel, or ribonucleotide inhibitors, such as pyrazofurin or cyclopentenylcytosine, have also been indicated to be dependent on a p53 pathway associated with activation or stabilisation of p53 RNA or protein (Linke *et al*, 1996; Gartel and Tyner, 1999).

Regulatory sites of STAT family transcription factors, the steroid nuclear receptor family including androgen receptor and vitamin D receptor, are also found within the promoters of the human p21 gene (Gartel and Tyner, 1999; Lu *et al*, 2000). The promoter between −119 and the start site of the transcription of the human p21 gene contains six Spl regulatory sites (termed as Spl-1 to Spl-6) and appears functionally different. Several important biological modifiers have been shown to activate p21 transcription through different Spl binding sites (Cartel and Tyner, 1999). For example, phorbol ester and okadaic acid induce p21 expression through Spl-1 and Spl-2 sites (Biggs *et al*, 1996), whereas the Spl-3 site in the promoter of p21 has been shown to be required for p21 induction by transforming growth factor-*β*, histone deacetylase inhibitors such as TSA and butyrate, lovastatin, nerve growth factor (NGF) as well as calcium (Datto *et al*, 1995; Nakano *et al*, 1997; Prowse *et al*, 1997; Sowa *et al*, 1997; Lee *et al*, 1998; Billon *et al*, 1999). In this report, we demonstrated that Spl-3 and Spl-4 in the promoter of human p21 gene are required for vinorelbine-mediated transcriptional restoration of p21 in the p21-deficient Al cells, which may provide a new mechanism in drug-mediated p21 regulation.
